# Microbial ecology and biogeochemistry of continental Antarctic soils

**DOI:** 10.3389/fmicb.2014.00154

**Published:** 2014-04-09

**Authors:** Don A. Cowan, Thulani P. Makhalanyane, Paul G. Dennis, David W. Hopkins

**Affiliations:** ^1^Department of Genetics, Centre for Microbial Ecology and Genetics, University of PretoriaPretoria, South Africa; ^2^School of Agriculture and Food Sciences, The University of QueenslandBrisbane, QLD, Australia; ^3^School of Life Sciences, Heriot-Watt UniversityEdinburgh, UK

**Keywords:** antarctica, microbial ecology, soil, hypoliths, nitrogen, carbon, adaptation, threats and impacts

## Abstract

The Antarctica Dry Valleys are regarded as the coldest hyperarid desert system on Earth. While a wide variety of environmental stressors including very low minimum temperatures, frequent freeze-thaw cycles and low water availability impose severe limitations to life, suitable niches for abundant microbial colonization exist. Antarctic desert soils contain much higher levels of microbial diversity than previously thought. Edaphic niches, including cryptic and refuge habitats, microbial mats and permafrost soils all harbor microbial communities which drive key biogeochemical cycling processes. For example, lithobionts (hypoliths and endoliths) possess a genetic capacity for nitrogen and carbon cycling, polymer degradation, and other system processes. Nitrogen fixation rates of hypoliths, as assessed through acetylene reduction assays, suggest that these communities are a significant input source for nitrogen into these oligotrophic soils. Here we review aspects of microbial diversity in Antarctic soils with an emphasis on functionality and capacity. We assess current knowledge regarding adaptations to Antarctic soil environments and highlight the current threats to Antarctic desert soil communities.

## Antarctic desert soils

**Figure 1:**
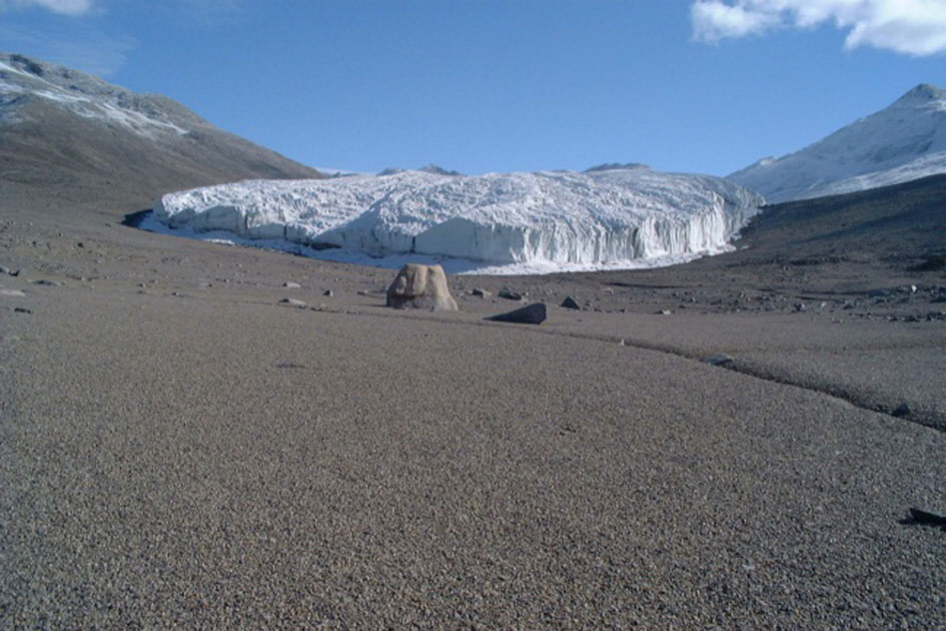
**Low altitude maritime soils in the McMurdo Dry Valleys**.

**Figure 2:**
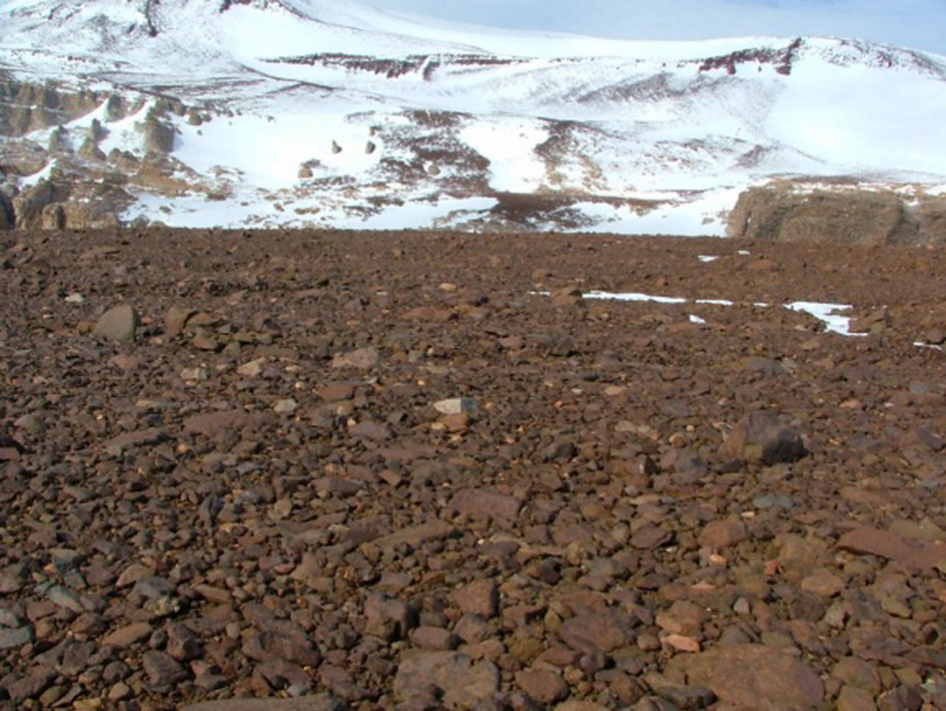
**Rocky soils of the high altitude Beacon Valley**.

**Figure 3:**
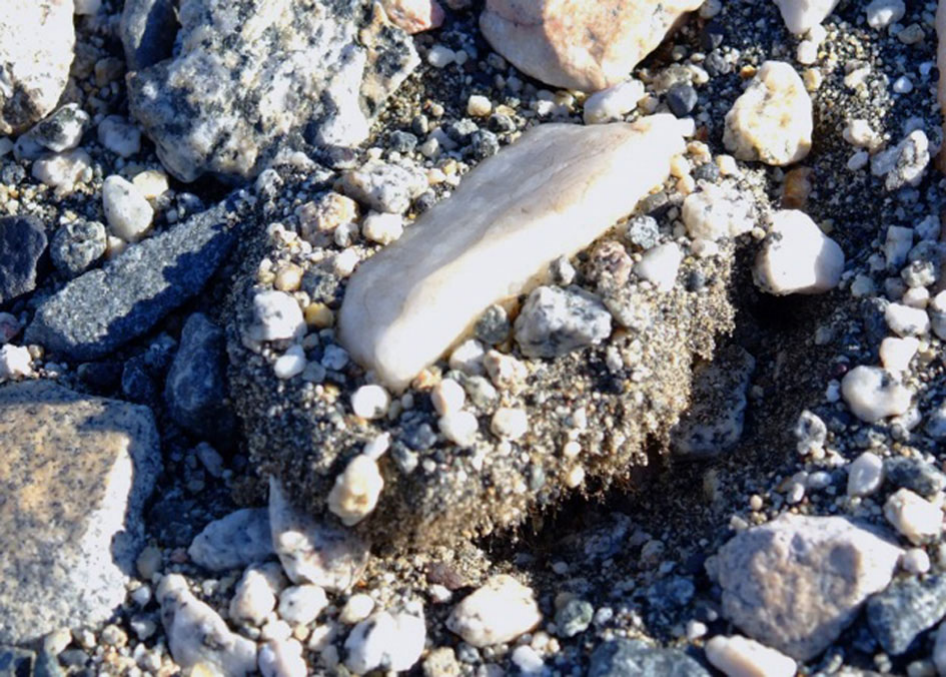
**The cryptic hypolithic habitat; a moss community associated with a quartz pebble**.

**Figure 4:**
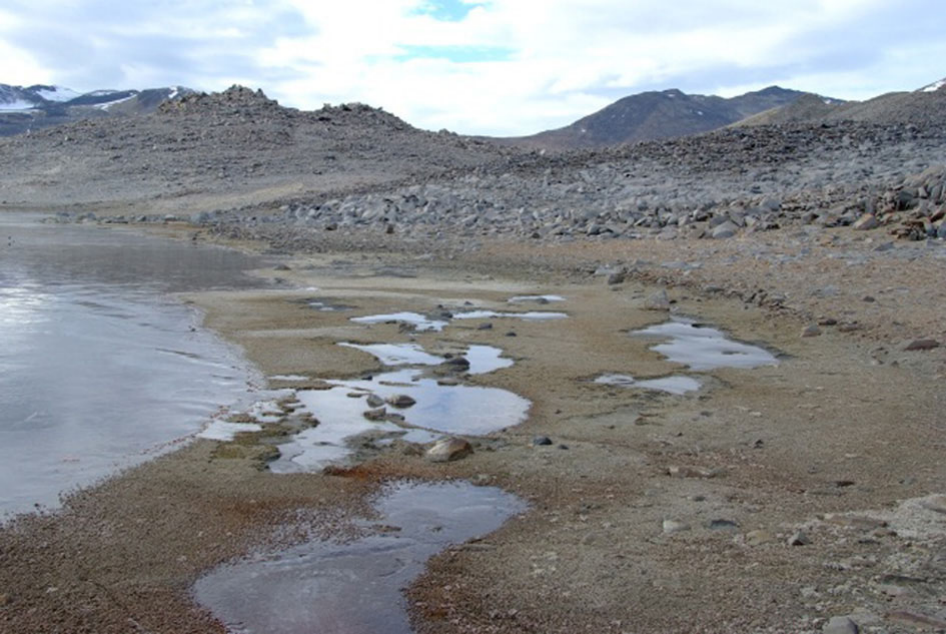
**Saturated soils and cyanobacterial mats associated with a shallow Dry Valley lake**.

**Figure 5:**
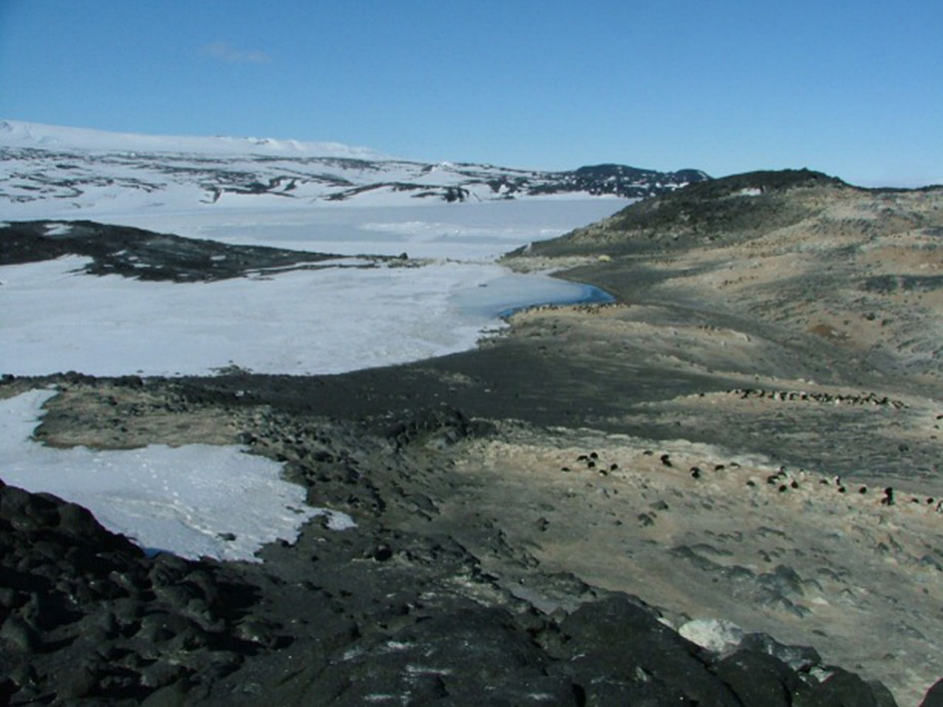
**Ornithogenic soils of an Adélie Penguin colony**.

**Figure 6:**
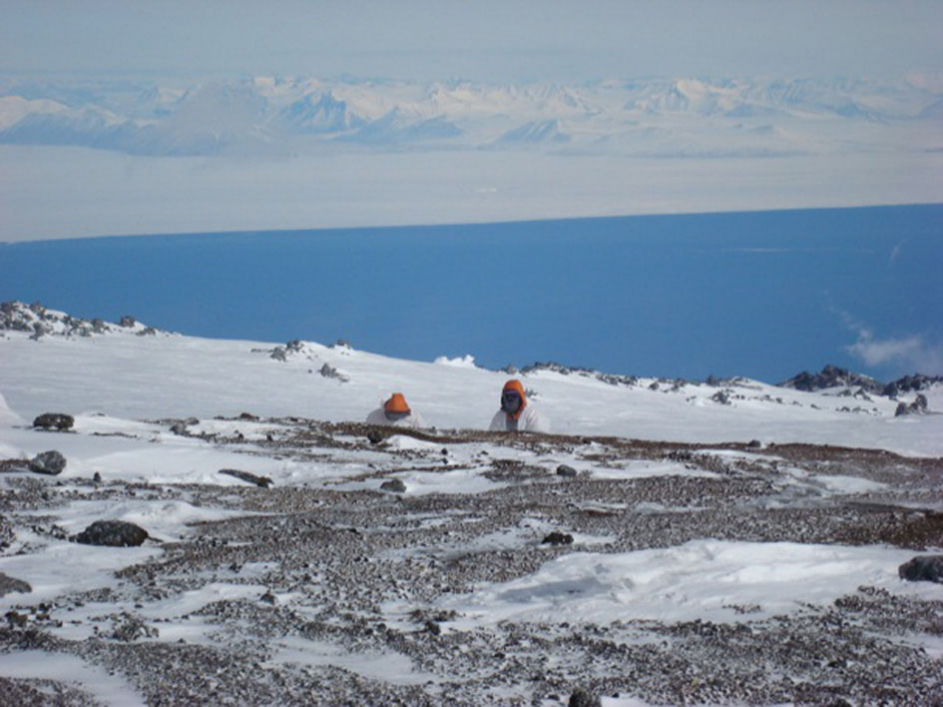
**High altitude thermally heated soils near the summit of Mt Erebus**.

**Figure 7:**
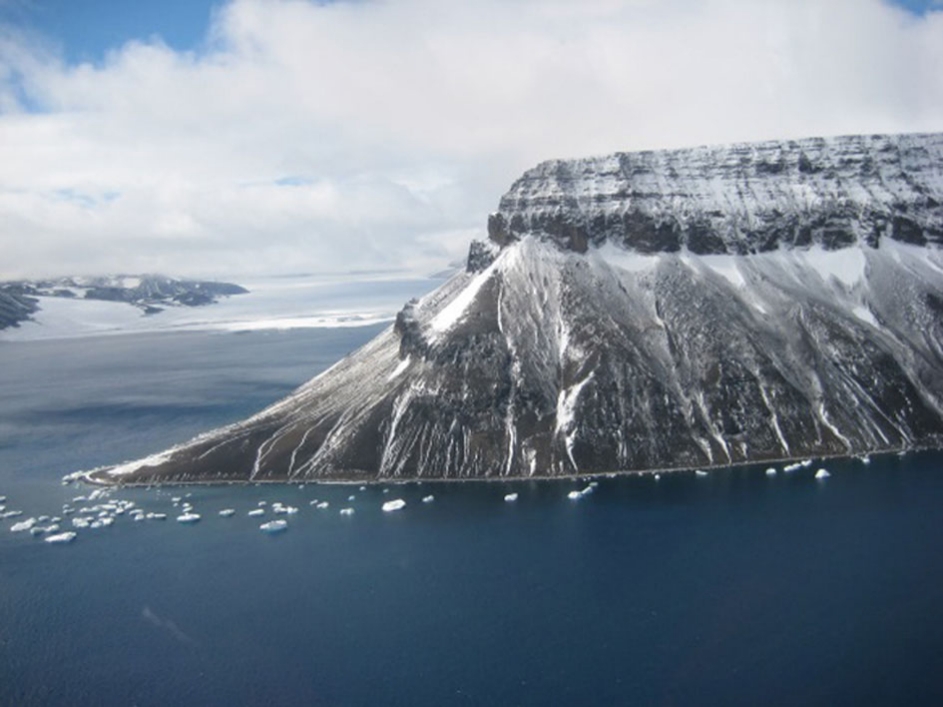
**The northern tip of that Antarctic peninsula where the soils receive liquid water from meltwater and summer rain**.

**Figure 8:**
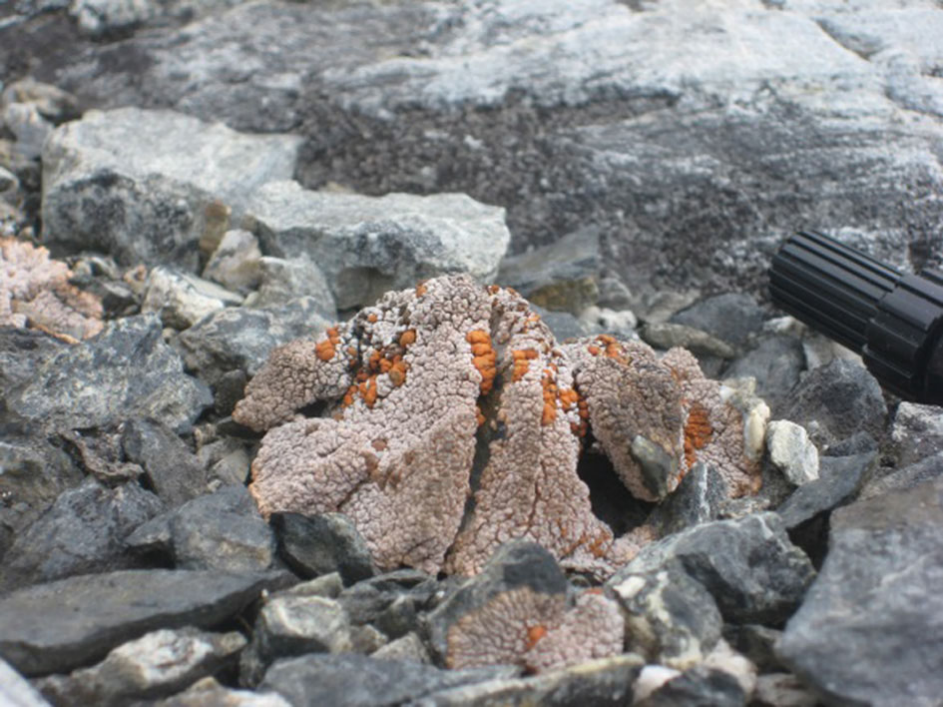
**Mixed lichen communities (including genus *Alectoria*) at the north of the Antarctic peninsula (Alectoria Island)**.

**Figure 9:**
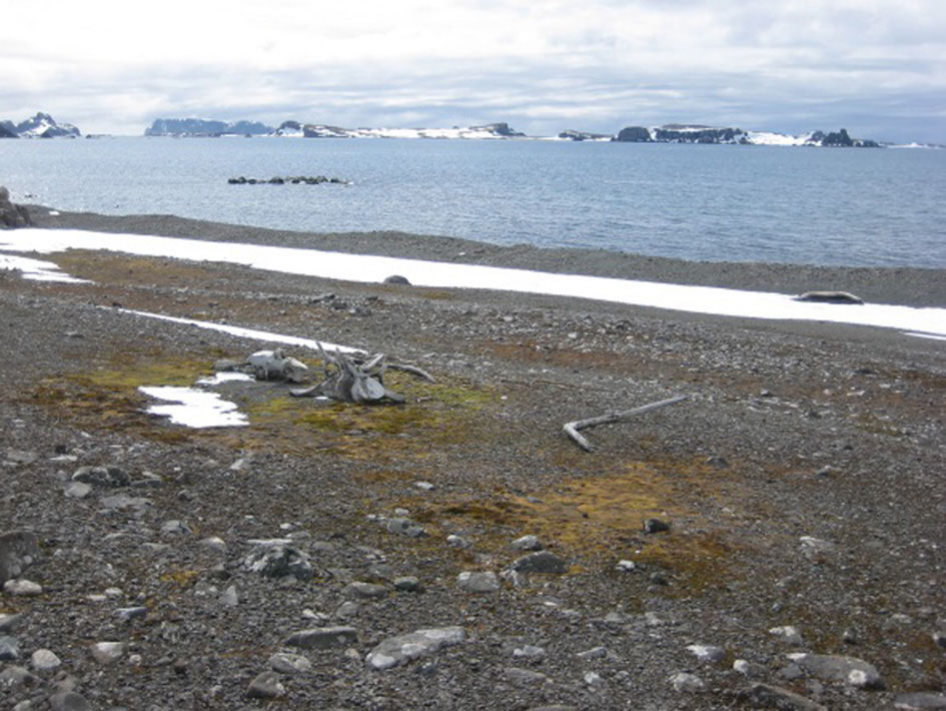
**Moss and algal communities associated with resources from whale bones (Livingston Island)**.

**Figure 10:**
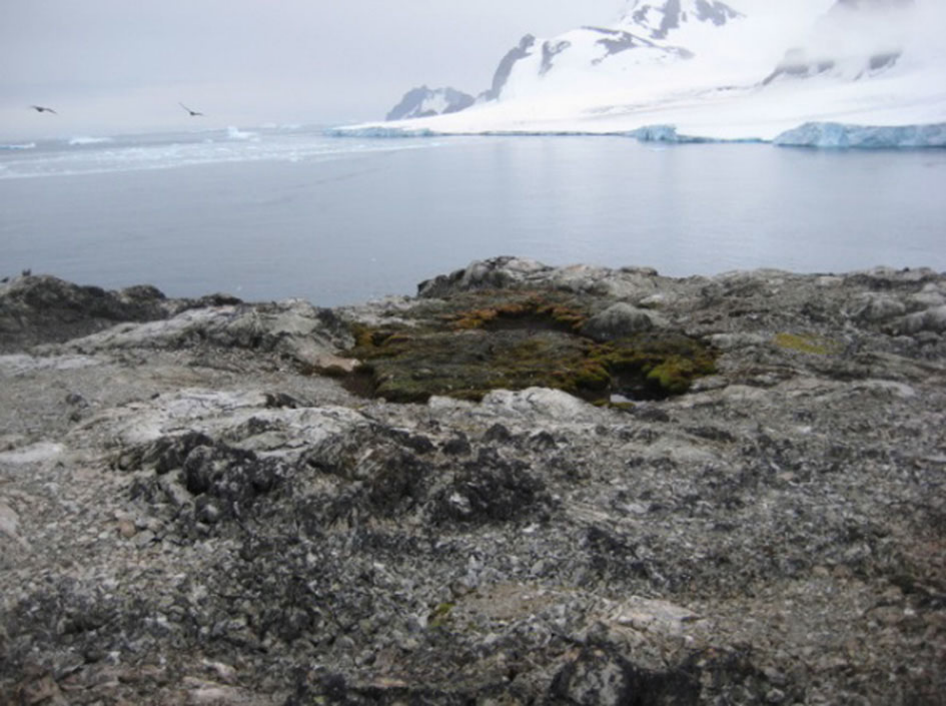
**The southern limit of flowering plants and peat growth at Lazarev Bay, north west of Alexander Island (69°22.0's; Convey et al., 2011)**.

Despite the fact that the ice-free areas of the Antarctic continent represent less than 0.3% of the total land area, the continent offers a wide range of different soil types, chemistries and microenvironments. This reflects such variables as the complex geological and glacial history of the continent. The wide variations in the landform structure such as the relatively low latitude Antarctic peninsula, the high latitude Dry Valleys, coastal valley systems and exposed sub-Antarctic mountains, coastal regions impacted by mammals and birds, and even high montane thermally heated soils, together provide the continent with a very wide range of soils and soil types. Figures 1–10 show examples of the very wide diversity of Antarctic edaphic habitats. The physicochemical parameters and environmental conditions of these diverse soils are equally wide-ranging (Bockheim and Ugolini, 1990; Bockheim and McLeod, 2008).

## Antarctic terrestrial “soil” as a microbial habitat

The Soil Science Society of America (2008) defines a soil as either “The unconsolidated mineral or organic material on the immediate surface of the Earth that serves as a natural medium for the growth of land plants” or “the unconsolidated mineral or organic matter on the surface of the Earth that has been subjected to and shows effects of genetic and environmental factors of: climate (including water and temperature effects), and macro- and microorganisms, conditioned by relief, acting on parent material over a period of time.”

While part of this definition is distinctly *phyllocentric*, it broadly encompasses the diverse range of Antarctic terrestrial “soil” habitats, despite the complete absence of higher plants in all areas other than the Antarctic Peninsula. Even the most extreme and apparently depauperate ice-free zones of the Antarctic continent can reasonably be considered as soil habitats, given that all contain microbial populations and detectable, if low, levels of organic carbon.

For a continent that is often considered to be little more than an expanse of ice, the presence of oligotrophic, copiotrophic, hyper-arid, water-saturated, permanently frozen (psychrophilic), and locally heated (thermophilic) soils offers scope for an equally diverse range of microbial physiologies and phylotypes (Gilichinsky et al., 2007; Niederberger et al., 2008; Stomeo et al., 2012; Bajerski and Wagner, 2013; Chan et al., 2013; Wilkins et al., 2013; Goordial and Whyte, 2014).

## Microbial diversity

The past two decades have seen a complete revolution in our understanding of the microbial diversity of Antarctic soil habitats. Early culture-dependent studies clearly demonstrated the presence of microbial endemism at the species (and to a lesser extent, the genus) level, but Antarctic soil isolates were restricted to a relatively narrow range of Families, dominated in particular by the Gram-positive Firmicutes (Vishniac, 1993). Hindsight now shows that Antarctic soil isolation studies, as for so many other terrestrial habitats, principally targeted the so-called “microbial weeds”; the fast-growing heterotrophic organisms for which the relatively rich culture media typically employed were not toxic.

**Figure 11:**
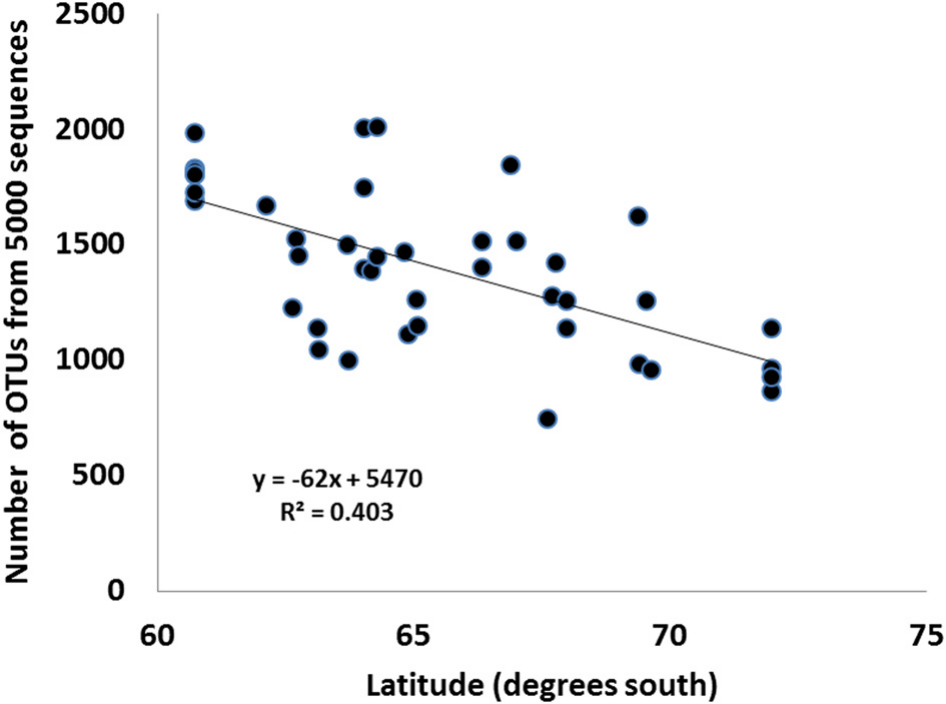
**Relationship between the number of operational taxonomic units (OTU) for 5000 16S rRNA gene sequences and latitude for soil samples from the maritime Antarctica**.

The introduction of modern molecular phylogenetic and metagenomic methods, which provide a much more comprehensive analysis of soil microbial diversity, has provided some surprising results. The first was that microbial diversity was much broader than ever suggested from culture-dependent analyses, with many of the clades identified also found in other (non-psychrophilic) soil habitats (Aislabie et al., 2006; Smith et al., 2006; Cary et al., 2010). By inference (i.e., inferring physiological characteristics from taxonomic identities), the physiological diversity of Antarctic soil organisms is equally wide (Chan et al., 2013), with many groups identified for which there were, at that stage, no cultured psychrophilic representatives (such as the Acidobacteria and the Verrucomicrobia). Secondly, it has become apparent that prokaryotic diversity indices from soils at high latitudes may not necessarily conform with the accepted trends typical for higher organisms (higher latitudes = lower diversity). Data from the east Antarctic Latitudinal Gradient Project (Howard-Williams et al., 2010), where diversity surveys were conducted from Cape Hallett (72°S) to the Darwin Glacier (84°S), show that there were no consistent latitudinal trends, and the local environmental parameters completely dominated larger spatial variables. Similarly, Dennis et al. (2012) report that soil fungal composition does not alter along a latitudinal gradient through the maritime and sub-Antarctic. However, the number of microbial diversity studies is still relatively small and it is too early for a full understanding of the pattern of microbial diversity under extreme conditions to be formed. The fact that conflicting results are reported clearly illustrates that our understanding of the drivers of soil microbial biodiversity in high latitude soils is currently unclear. For example, in the Antarctic, Howard-Williams et al. (2010) and Dennis et al. (2012), and in the Arctic, Neufeld and Mohn (2005) report no clear trend of microbial diversity with latitude, whilst Yergeau et al. (2007a,b) and Dennis et al. (manuscript submitted for publication) report clear trends of declining bacterial diversity in the maritime Antarctic (Figure 11). The possible drivers for such a latitudinal decline in diversity are currently unknown, although mean temperature, and the secondary effects of temperature on water activity, are obvious candidates.

**Figure 12:**
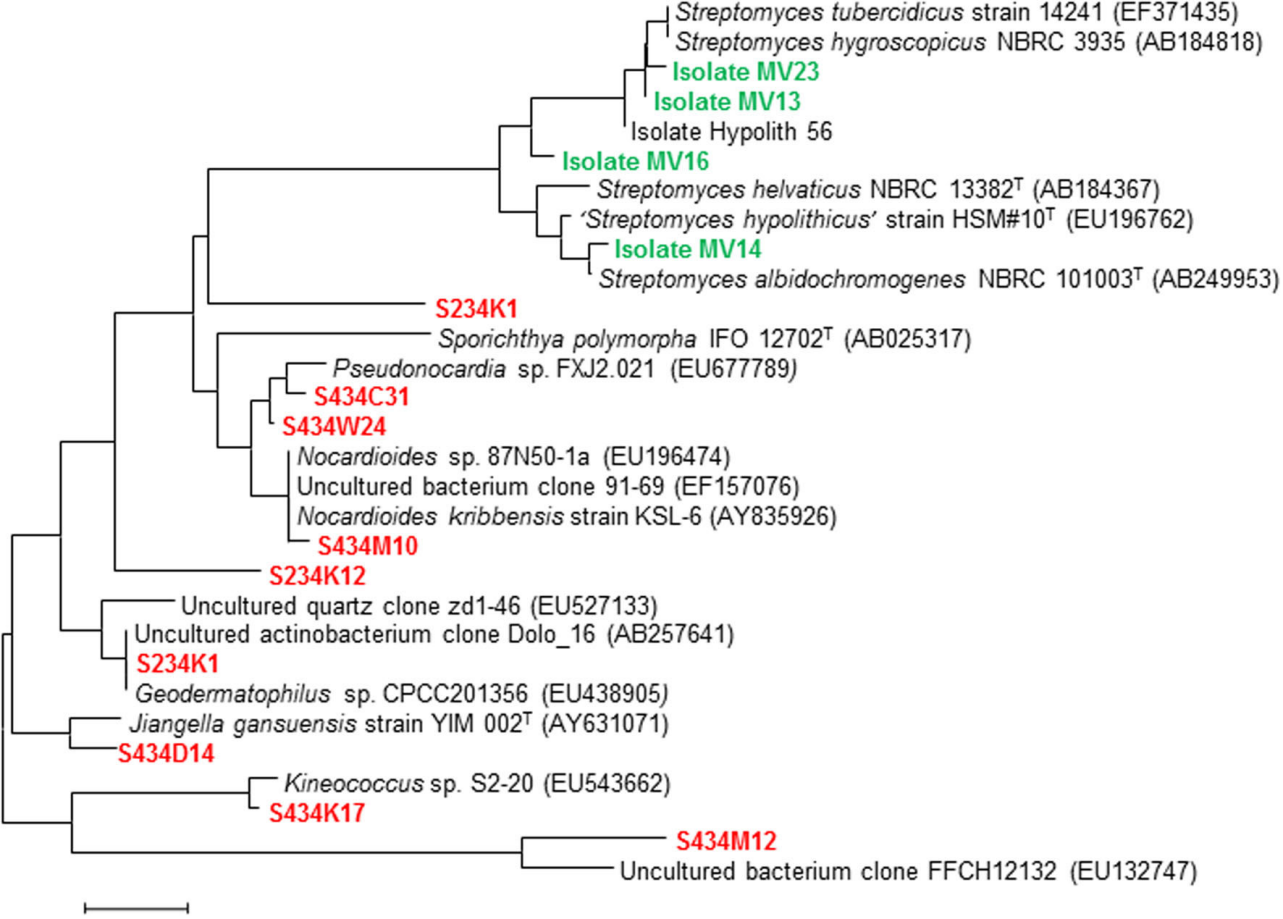
**Phylogenetic distribution of Actinobacteria in Antarctic Dry Valley soils (clones in red: redrawn from Babalola et al., 2009), indicating cultured taxa (isolate codes in green: Cowan et al., unpublished results)**.

As is typical of other soil habitats, certain taxa dominate most Antarctic soil phylotypic datasets, the metabolically diverse Proteobacteria and the Actinobacteria being the most prominent (Babalola et al., 2009; Makhalanyane et al., 2013). The prevalence of the latter, particularly the presence of numerous uncultured representatives of the filamentous Actinomycetes, is particularly interesting from a biotechnological perspective, since this group of bacteria has contributed a high percentage of the world's antibiotics. As is now well engrained in the perception of microbiologists, success in phylotypic identification is generally not paralleled by success in accessing these species by classical culturing (e.g., Figure 12).

One of the surprising discoveries from the surveys of Antarctic soils has been that while bacterial diversity is high, both fungal and archaeal diversities are unexpectedly low, at least in arid and hyper-arid soils (Pointing et al., 2009; Rao et al., 2012). The identification, in soil samples from the inland McKelvey Valley, of only 7 fungal phylotypes, two of which were affiliated to known genera (*Helicodendron* and *Zalerion*), suggests that free-living fungi may be more susceptible to environmental extremes than bacteria. It has also been suggested that the low fungal diversity might be the result of dispersal limitations (Rao et al., 2012). However, recent aerobiological surveys of bacterial and fungal spore transport in coastal Antarctica (Duncan et al., 2010; Bottos et al., 2014), indicate that viable cells (and spores) are transported in substantial numbers and potentially over very large distances.

It is also notable that Antarctic desert surface soils and soil niche habitats, which are typically aerobic, show few, if any, archaeal taxa, and it is possible that Archaea play a minimal role in soil processes (Pointing et al., 2009; Ayton et al., 2010; Khan et al., 2011). However, it should be noted that very few Archaea-specific phylotypic surveys of Antarctic soils have been reported and, coupled with the technical difficulties of culturing psychrophilic methanogens and chemoautotrophs, it is reasonable to conclude that the exact role of Archaea in edaphic habitats has yet to be resolved.

What has also become clear is that microbial diversity is anything but homogeneously distributed, even in habitats that appear, at least at an observational level (such as the Dry Valley deserts) as homogeneous. A recent and very revealing phylogenetic survey of soil samples from a number of “geographically disparate” Antarctic Dry Valleys (Lee et al., 2012), shows very distinct regional clustering, with only “2 of 214 phylotypes found in all four valleys.” The significance of this dramatic observation has yet to be fully appreciated, but may become a key factor in helping to understand the drivers of diversity, and in guiding future conservation planning strategies.

It is very evident that a current major gap in our understanding of the composition of Antarctic soil (and many other) communities are the role of viruses and phages. Knowledge of the terrestrial virome of Antarctica is far from complete and even where there is some information it is fragmentary and frequently arising from incidental observations and opportunistic investigations (Hopkins et al., 2014). To date, only a single systematic Antarctic soil phage survey, of saturated Antarctic Peninsula soils, has been published (Srinivasiah et al., 2008). This study reported phage densities in the order of 2 – 6 × 10^8^ g^−1^, only 2- to 10-fold lower than in temperate agricultural and forest soils, respectively. The occurrence of apparently unusual observations, such as the “Russian doll” viruses with two genomes in the same phage (Swanson et al., 2012), virophages (Yau et al., 2011) and the large phage-to-bacteria ratios (Williamson et al., 2007) in Antarctica may point to the novelty of the extreme environments, but with such a small number of studies so far conducted further speculation is unwarranted.

There is strong evidence to suggest that most soil bacteriophages exhibit a lysogenic rather than a lytic lifestyle (Williamson et al., 2007), and are therefore largely missed using standard soil extraction and concentration techniques. Single- and double stranded RNA viruses of lower eukaryotes and some prokaryotes would also not be detected by standard DNA extraction protocols. Given the known numerical dominance of bacteriophages in many ecosystems (Suttle, 2007; López-Bueno et al., 2009; Fancello et al., 2013), and their key roles in controlling microbial populations and biogeochemical cycling (Weinbauer, 2004; Laybourn-Parry, 2009), the paucity of studies on terrestrial Antarctic “virology” represents a gap in our understanding of the composition and functioning of Antarctic soil communities.

## Carbon and nitrogen cycling

Given the extreme nature of many Antarctic soil environments, with their low nutrient status, very low mean temperatures and relatively short periods when the combination of temperature and water availability positions the extant communities in the metabolically active zone, it is widely assumed that the contributions of such communities to systems processes (such as C and N turnover) are minimal, and that the communities themselves are stable over long time periods. However, there is growing evidence that neither of these assumptions are necessarily well grounded. Various *in situ* and *ex situ* functional analyses, including isotope labeling studies and high resolution respirometry, suggest that core functions are maintained to well below 0°C, and that niche communities such as hypoliths may be important contributors to landscape-scale processes such as dinitrogen fixation.

The rate of change of community composition (as judged using fingerprinting methods such as (*Automated) Ribosomal Intergenic Spacer Analysis* (ARISA) which monitor the dominant 20–40 phylotypes in a sample) may be much higher than previously thought. A simple but elegant experiment involving the physical repositioning of a ca. 200 year old seal carcass from one site to another clearly demonstrated a complete restructuring of the underlying soil community within a 3 year period (Tiao et al., 2012). Irrespective of the exact driver(s) of this change, whether temperature-, water-, light-, or nutrient-dependent, the rapid timescale of the change is unequivocal.

Antarctic soils have been shown to harbor the genetic capacity for both autotrophic carbon fixation and nitrogen fixation (Hopkins et al., 2006a; Cowan et al., 2011b; Cameron et al., 2012; Niederberger et al., 2012). This genetic capacity has been shown for a range of edaphic Antarctic environments, ranging from lacustrine microbial mats, cryptic, and refuge niches (hypoliths and endoliths) to superglacial cryoconite communities (Hopkins et al., 2006a; Severin et al., 2010; Cowan et al., 2011b; Cameron et al., 2012; Chan et al., 2013). What remains unclear, however, is whether these capacities represent historical signatures (i.e., “legacy fingerprints”) from previous microbial contributions, or whether they represent ongoing soil processes. Enzyme activities are a useful indicator of potential microbial activity and may provide some indication of the metabolic range of the soil (Hopkins et al., 2008). A study by Hopkins et al. (2006a,b) focused on soil respiration in the Garwood Valley suggests that lacustrine detritus, emanating from the lake shore, may be an important driver of soil respiration in the Antarctic Dry Valley soils. Nitrogen cycling processes have been investigated through application of acetylene reduction assays (Cowan et al., 2011b; Niederberger et al., 2012). In ephemerally wetted soils *in situ* N_2_ fixation assays and RNA-based quantitative PCR assays showed rates between 0.5 and 6 nmol N cm^−3^ hour^−1^. These measurements indicated that up to half of this activity, linked to a range of diverse phyla, mainly proteobacteria and cyanobacteria, associated with sulfate reduction. Hypolithic microbial communities have been shown to possess the genetic and functional capacity for nitrogen fixation (Cowan et al., 2011b). The total contribution of hypolithic communities to the Miers Valley region (11 km in length and between 1.5 and 2.5 km wide Cowan et al., 2010) was estimated at 14,200 mmol N year^−1^ (0.38 kg N year^−1^) indicating the significant contribution of these communities to fixed nitrogen budgets in Antarctic desert soils. A direct interpretation of these data suggests that hypoliths may contribute significantly to the total carbon and nitrogen turnover in cold desert ecosystems (Hopkins et al., 2009; Cowan et al., 2011b).

A seminal study by Yergeau et al. (2007a,b) employed a multifaceted approach using real-time PCR, enzymatic assays, process rate measurements and functional microarrays in order to deconvolute the environmental factors driving soil N and C-cycling in terrestrial Antarctic ecosystems [from 51°S (Falkland Islands) to 72°S (Coal Nunataks)]. The abundance and diversity of functional gene families was found to differ significantly with temperature, sampling location and vegetation type. Interestingly, microbial C-fixation genes were more abundant in plots lacking vegetation. Denitrification genes dominated in the higher temperature soils and nitrogen fixation genes were prevalent in lichen-vegetated sample sites (Yergeau et al., 2007a).

The rapid warming of the continent has been shown to affect bacterial communities more than fungal communities (Yergeau and Kowalchuk, 2008). It is known, for instance that freeze thaw cycles (FTC) have been reported to affect key steps of the N-cycle, while FTC and temperature have been shown to influence laccase enzymatic activity in Antarctic soils (Yergeau and Kowalchuk, 2008). These results suggest that in addition to climatic warming, increased climatic variability may impact microbial communities in Antarctica.

## Adapting to the antarctic soil environment

It is accepted without question that survival in the Antarctic environment, by any organism, is a consequence of both ecological selection and evolutionary adaptations which are expressed at the behavioral, physiological, metabolic, structural, and genetic levels. As an example of behavioral adaptation, Antarctic springtails (such as *Neocryptopygus* sp.) are found most commonly on the ventral surfaces of thin dark rock shards where the surface adsorption of solar radiation presumably elevates the temperature of the underside and increases the potential for mobility and grazing behavior (Caruso et al., 2009). An example of ecological selection might be the propensity for the development of endolithic and hypolithic microbial communities in Antarctic ice-free areas where suitable translucent minerals (most commonly sandstone, marble and quartz) are present. In such niche habitats, it is apparent that a combination of factors, including access to Photosynthetically Active Radiation (PAR) and improved moisture availability, and avoidance of desiccation and physical disturbance, are the driving forces for development (Cary et al., 2010; Chan et al., 2012).

At the cellular and sub-cellular levels, much of our understanding of the physiological and molecular adaptations of Antarctic (soil) microorganisms to their unique environment comes from culture-dependent studies. While adaptations at the genetic and cellular level are not the focus of this text, and some have been comprehensively reviewed elsewhere (Feller and Gerday, 2003; Siddiqui and Cavicchioli, 2006; Casanueva et al., 2010), by far the greatest emphasis has been on thermoadaptation (Graumann and Marahiel, 1996; Goodchild et al., 2004; Médigue et al., 2005). The evolutionary responses of microorganisms to the potentially damaging consequences of frequent freeze-thaw events, loss of membrane fluidity and the impacts of Arrhenius Law are reasonably well understood (Hoyoux et al., 2004). The expression of cold shock proteins, the biosynthesis of compatible solutes, the use of thermal hysteresis and ice nucleation proteins (Guerrero et al., 2014), membrane lipid thermoadaptation and changes in protein amino acid composition are all examples of adaptation to the thermal conditions prevalent in cold environments (Khan et al., 2011).

Despite this depth of understanding, the molecular and physiological responses to other Antarctic soil stress elements, such as short wavelength ultraviolet (UV) radiation, desiccation and low light flux, have received relatively little attention (Hughes et al., 2003; Cowan et al., 2010).

Phylogenetic surveys of Antarctic soil prokaryote diversity suggest that certain clades known for their high levels of resistance to desiccation and radiation stress (such as the *Deinococcus-Thermus* clade) are possibly more prevalent than in less “hostile” soil habitats (Soo et al., 2009; Cary et al., 2010). However, the molecular and genetic basis for such resistance has, at least in psychrophiles, been subject to limited research (reviewed extensively by Casanueva et al., 2010). There is clearly considerable scope for using a “systems biology” approach, including the parallel application of genomics, transcriptomics, proteomics and metabolomics, to study and understand the basis of adaptation and stress response in single organisms. With the rapid growth of very high throughput, high resolution analytical systems (Next Generation DNA and RNA sequencing of metagenomes and metatranscriptomes, LC-MS analysis of metaproteomes and meta-metabolomes, microarray systems), it can be confidently predicted that the next decade will see a dramatic rise in whole population analyses, giving detailed information on the range and role of different stress response systems in entire microbial communities rather than the adaptations of single species.

## Threats and impacts

The Antarctic continent, and particularly the exposed and therefore sensitive soil ecosystems, are perceived to be under threat from two unrelated but potentially substantial impacts: climate change (Walther et al., 2002) and direct human impact (Cowan et al., 2011a). That climate change is already affecting the continent is irrefutable (Petit et al., 1999; Doran et al., 2002; Walther et al., 2002; Vaughan et al., 2003; Jung et al., 2011; Yergeau et al., 2011). High resolution terrestrial climate data from over many decades show a dramatic rise in the temperatures of most coastal regions of the Antarctic continent, with the Antarctic peninsula being most substantially affected. Due to the limited impact of the circulating waters of the Antarctic current, the Ross Sea area, which coincidentally harbors much of the ice-free area of the continental surface, is the only region not showing a significant rise in temperature. These rises in temperature have caused melting of buried and surface ice, which has led to wetting of previously dried soils. The increased moisture is likely to alter soil microbial communities by mobilizing nutrients and salts, completely changing the carbon input dynamics in soil (Simmons et al., 2009). Recent studies have reported an increase in solar radiation over the McMurdo Dry Valleys of Eastern Antarctica (Turner and Overland, 2009).

The extent, and implications, of direct human impacts are much less clear, but no less a cause for concern. As detailed by several recent reports (Hughes and Convey, 2010; Cowan et al., 2011a), the steady rise in both tourist and research-related activities increases the pressure on the most sensitive habitats of the continent. Physical impacts (chemical spillages, disturbance to sensitive animal and “plant” communities) are readily observed and measured, and are therefore controllable. Other impacts are less obvious and less well understood. There is a growing realization that the presence of human activity leaves a non-indigenous “footprint,” in the form of macroscopic propagules (seeds) and microscopic entities (bacteria and human cellular material). While the former is readily avoidable through the use of good screening and decontamination practices, the latter is virtually impossible to control without imposing highly restrictive operational controls. Although such microbial contaminants may not retain viability in the more extreme soil areas of the continent, such as the Dry Valleys, they are likely to leave a substantial genetic fingerprint (Cowan et al., 2011a). Given the prevalent conditions of Antarctic soil environments (cold and dry); these fingerprints may be very long-lived. Virtually nothing is known of the consequences of this non-indigenous biological input, the mobility and transport of the contaminants, their long-term stability, the quantitative and qualitative consequences of horizontal gene transfer, or the consequence of any of these in terms of ecosystem functioning.

## The way forward

The soil habitats of the Antarctic continent are diverse, in many ways unique, have highly restricted dimensions on a global scale and offer huge scope for research into fundamental issues of ecosystems ecology and adaptation. These habitats are not static, to the extent that they have been hugely impacted by the glacial history of the continent, but such changes have been on a millennial scale. The recent and relatively rapid warming in parts of Antarctica, notably the Antarctic Peninsula, will undoubtedly lead to more rapid changes in the structure and function of edaphic microbial communities, principally in response to the likely increase in the availability of substrates and liquid water. However, the current limitations in our understanding of the controls on the activity of the terrestrial microorganisms, and our even poorer understanding of the interactions between species in complex microbial communities, means that we are not well positioned to be able to predict exactly how such communities will respond to change. We suggest that more extensive research, relating variations in the ecological fitness of individual microbial guilds to population dynamics and community composition and function are required. The comparatively simple trophic structures and (presumed) long term stability of Antarctic communities and habitats provide an ideal basis for these studies. A determination of the different strategies used by microbial communities and individual species to maximize their chances of survival is crucial for understanding both ecosystem processes and the biogeochemical feedbacks to climate change (Nie et al., 2013; Evans and Wallenstein, 2014). The Antarctic continent has been subject to huge climate shifts over geological time. Pressure from human activities is, by comparison, a very short term effect, in that human occupation of the continent is restricted to little more than a century and is most significant over the past half century. We are yet to fully comprehend how human activities will impact both the structure and functioning of edaphic microbial communities in Antarctica and how these activities may, in the future, impact on our abilities to study these communities. Given the recent rapid growth in the scope and sensitivity of phylogenetic and metagenomic methodology, and projecting a similar trajectory over the next few decades, one might project a time when the “contaminant” signals in Antarctic soils become a significant barrier to accurate assessments of the true microbial diversity.

The early twenty-first Century has seen a growing awareness of the threats to the Antarctic continent. Recent interventions, such as the 2012 50-year Antarctic Horizon Scanning meeting (Chown et al., 2012) and the 2014 SCAR^1^. -sponsored Antarctic and Southern Ocean Horizon Scan (http://www.scar.org/horizonscanning/), were aimed at understanding current issues and threats, and predicting both their evolution and their consequences. The outputs of these deliberations can, at least in theory, be channeled through SCAR, as the international body responsible for Antarctic science policy, to the Antarctic Treaty nations. This process can, as it has done in the past, ultimately influence national and international policies on activities on and around the Antarctic continent.

The clear message, from a survey of the current state of knowledge on Antarctic soil microbiology, is that much more focused research is required. Despite a growing compendium of comprehensive phylotypic surveys, basic questions relating to local and regional species heterogeneity, and to the impacts of the macro- and microenvironment on the structure of microbial communities remain essentially unanswered. Even less is known of the *in vivo* functions of these communities, and how these processes respond to short- and long-term changes. The application of metagenomic and metaproteomic approaches has already provided valuable insights into biological functionality in Antarctic marine environments (Peck et al., 2004; Williams et al., 2012; Yau et al., 2013), and could equally be applied in terrestrial environments in order to understand these systems. While there has been a steady increase in the number of publicly available Antarctic bacterial, archaeal, and virus genomes (Allen et al., 2009; Carneiro et al., 2012; Meiring et al., 2012; Park et al., 2012; Morita et al., 2013), we are yet to fully understand the relationships between genome potential and biological function, with in individual organisms or complex microbial communities. Valuable insights into the metabolic potential of microbial communities have come from recent gene-specific surveys and Geochip® analyses, but the accurate quantitation of *in vivo* metabolic rates remains both a substantial challenge and a very important objective.

## Attributions

Figures 1–5, Don A. Cowan; Figure 6, Craig S. Cary; Figures 7–12, David W. Hopkins; Figure 10, Peter Convey.

### Conflict of interest statement

The authors declare that the research was conducted in the absence of any commercial or financial relationships that could be construed as a potential conflict of interest.

## Footnotes

^1^SCAR: The Scientific Committee on Antarctic Research (http://www.scar.org/)
